# Requirements for patient-reported outcomes and data analytics in health technology assessment in England, France and Germany, and the need for methods harmonization across European markets: a qualitative interview study

**DOI:** 10.1186/s41687-026-01059-4

**Published:** 2026-04-03

**Authors:** Laurie DiModica, On Yee Wong, Sarah Knight, Melanie Calvert, Olalekan Lee Aiyegbusi, Saeid Shahraz, Yanni Hao, Denise Globe

**Affiliations:** 1 Clarivate (United States), Boston, USA; 2 Clarivate, London, UK; 3 University of Birmingham, Birmingham, UK; 4 Gilead Sciences (United States), Foster City, USA

**Keywords:** Patient-reported outcomes, PROs, PRO analytics, Health technology assessment, Joint clinical assessment, JCA

## Abstract

**Objectives:**

To explore methods for collection, reporting and analysis of patient-reported outcome (PRO) data in health technology assessment (HTA) in England, France, and Germany, and understand the impact and challenges associated with using PRO data in HTA.

**Methods:**

A targeted literature review (TLR) was conducted in November 2023 to identify PRO-related data specifications for HTA in European markets and inform an interview discussion guide. Qualitative, semi-structured 60-minute interviews with key opinion leaders (KOLs) with HTA- and PRO-related expertise in England, France and Germany were conducted to gain expert perspectives on PRO and HTA practices, and to identify opportunities for HTA harmonization across European markets. The interview transcripts were analyzed using content analysis methods and key results extracted by a single analyst into Microsoft^®^ Excel.

**Results:**

KOLs from England (*n* = 4), France (*n* = 3) and Germany (*n* = 5) were interviewed. Availability of guidance on PRO data collection, analysis and reporting in HTA varies between the HTA bodies included. Guidance, where available, is country specific, and therefore lacks harmonization across included markets. In terms of the perceived impact of PRO data on HTA decision making, KOLs from Germany gave a high rating, while KOLs from England and France gave ratings of low to high impact and moderate to high impact, respectively. It was reported by KOLs in all markets that PROs are expected for submissions in highly symptomatic and burdensome conditions, and that the relative importance of PRO to HTA outcomes varies by disease. KOLs cited challenges with using PRO data in HTA. Commonly cited challenges were related to methodological considerations and included: ‘no/suboptimal PRO data collection/submission (e.g., due to value perceptions)’ and ‘selected instruments not fit-for-purpose/PRO benefits not adequately represented in models’. Adding to the challenges with using PRO data in HTA, there are inconsistencies between Joint Clinical Assessment (JCA) guidance and PRO data requirements for HTA in included markets.

**Conclusions:**

There is a lack of transparency and harmonization in terms of the requirements for PRO data collection, analysis and reporting for HTA in England, Germany and France. Enhanced transparency of HTA requirements will facilitate harmonization efforts and may be supported by JCA.

**Supplementary Information:**

The online version contains supplementary material available at 10.1186/s41687-026-01059-4.

## Abstract (significance)

Why did we do this study?

What did we do?

What did we find?

What does this mean?

For reimbursement of new therapies, health technology assessment (HTA) bodies need to understand the impact of treatment on patients’ quality of life, symptoms, and daily functioning. This information comes from patient-reported outcomes (PRO) data. However, requirements for collecting and reporting this information for HTA processes can differ between regions. This lack of consistent guidelines creates uncertainty when developing new treatments and may delay patient access to new therapies.

Published guidance about PRO requirements for HTA from England, France, and Germany was reviewed. Semi-structured qualitative interviews were conducted with 12 experts with HTA- and PRO-related expertise in these countries. They were asked about current practices and challenges with using PRO data in HTA, and opportunities to make the requirements more consistent across countries.

The availability of guidance varies between England, France, and Germany. German experts reported that PRO data had a high impact on HTA decisions, whereas English and French experts reported more mixed impact, depending on factors such as the therapy area. Experts cited challenges related to methodological considerations, such as measurement tools that don’t capture what’s important to patients, and inconsistencies between European-level guidance and country requirements.

European health systems need clearer and more consistent requirements for using PRO data. This would improve understanding of HTA expectations when planning for HTA submissions, ensure patient voices are heard in HTA decisions, and may enable patient access to new treatments. The new European Joint Clinical Assessment process could help achieve this harmonization if country-specific PRO guidance is available and consistent.

**Supplementary Information:**

The online version contains supplementary material available at 10.1186/s41687-026-01059-4.

## Background

Patient-reported outcomes (PROs) are measures of symptom burden, physical and role function, and health-related quality of life (HRQoL) that come directly from the patient, to provide a patient-centered perspective on a disease or how well an intervention is working [1, 2]. PROs reflect the patient’s description of the impact of a disease or treatment on factors such as symptoms and physical, mental, and social well-being [2]. As well as capturing how a new treatment is impacting disease symptoms, PROs are increasingly used to assess the safety and tolerability of interventions [1].

In clinical trials, PROs complement standard clinical outcomes such as measures of survival and disease progression, and are widely used as primary, secondary or exploratory endpoints, depending on the disease area and treatment goals [1, 2].

Regulatory bodies routinely utilize PRO data along with clinical efficacy and safety endpoints to support decision making during the benefit-risk appraisal of new technologies [3, 4]. In health technology assessment (HTA), PRO data have historically not been considered key components for decision making, with the exception of the EQ-5D (EuroQoL-five dimensions). However, PRO data are increasingly being generated by health technology developers (HTDs) and used in HTA submissions as a way of capturing the patient perspective on the benefit of a new treatment [5]. In addition, PRO data are particularly important for demonstrating the value of treatments where the intention is not to cure, but rather alleviate symptoms and improve quality of life (QoL) [5–9]. Despite the growing recognition of the value of PRO data in HTA submissions among payers and HTA bodies, use of PRO data in clinical benefit assessment and health economic appraisal assessments is not clearly defined or consistent across HTA bodies [5, 6].

European Joint Clinical Assessment (JCA) aims to harmonize the clinical aspect of HTA for new treatments in EU member states [10]. JCA guidance issued in mid-2024 by the Member State Coordination Group on HTA Guidance provides parameters for member states to define relevant outcomes during the scoping process, but does not provide quantitative thresholds related to PRO data analytics [10]. The lack of explicit guidance on the requirements for PRO data analytics in HTA may make cross-market PRO data standards unlikely in the near-term. Furthermore, the requirements for PRO data in HTA are likely to vary across markets (for example, population, intervention, comparison and outcome [PICO] criteria are likely to be country-specific), which would impact the ability to achieve PRO data analytics harmonization.

If harmonization of the requirements for PRO data analytics in HTA is not achieved, HTDs face uncertainty around HTA expectations to guide their PRO strategy and analysis methods when planning for HTA submissions. As a result, HTDs may be prevented from coordinating clinical development and HTA strategies, which may negatively impact reimbursement and patient access to new treatments. Therefore, there is an urgent need for stakeholders to be transparent with methodological approaches to using PROs in HTA submissions. This could subsequently help to align methods, where appropriate, of PRO data usage across markets.

This qualitative study sought to capture and compare requirements for PRO and data analytics in HTA in England, France and Germany to understand methods for collection, reporting and analysis of PRO data in HTA. Semi-structured interviews aimed at gaining KOL perspectives on PRO and HTA practices were conducted.

## Methodology

### Targeted literature review

A targeted literature review (TLR) was conducted in November 2023 to inform the interview discussion guide and to identify key sources of information on PRO-related data specifications in HTA in European markets, including EU-level guidance on JCA (Member State Coordination Group on HTA [HTACG]) and methods guidance produced by national HTA bodies in three European markets (England [National Institute for Health and Care Excellence (NICE)], Germany [Institut für Qualität und Wirtschaftlichkeit im Gesundheitswesen (IGWiG) and Gemeinsamer Bundesausschuss (G-BA)] and France [Haute Autorité de Santé (HAS)]).

### TLR analysis and development of the interview discussion guide

Information from sources identified in the TLR was extracted into Microsoft Excel^®^ and categorized by theme, according to PRO data collection, analysis and reporting categories reflected in recent research [5]. Searches and data capture were conducted by a reviewer, with a second reviewer responsible for a quality check of the relevance of the extracted material and its categorization. Data were explored to inform assumptions on the existence of standards, level of transparency, and use of PRO data analytics in HTA, and used to develop questions for interviews with key opinion leaders (KOLs) experienced in PRO evaluations for HTA.

An interview guide was developed according to identified research themes based on a study of differences in PRO evidence requirements in oncology across key HTA bodies [5]. Specifically, the interview guide explored the following themes: (1) General integration of PROs in HTA, including HTA decision making and price setting, (2) PRO collection and reporting, including preferred instruments, missing data threshold and post-progression data collection, (3) PRO data analytics, including clinically meaningful change threshold, preference for time-to-endpoint definitions and missing data sensitivity analyses. In addition, KOLs were asked what they perceive the impact of PRO data on HTA and/or pricing outcomes to be.

### Recruitment

Potential interviewees were screened and recruited (with purposive sampling) for qualitative research based on pre-defined search criteria to ensure participants had relevant experience (see Appendix 1 for the participant screener and background details). KOLs were selected based on their knowledge, roles (either in academia or HTA bodies), and expertise, and had no prior relationship with the authors of this study. KOLs with expertise in PRO policy making or assessment of PRO data in HTA submissions from the last 5 years in England, France and Germany were selected and were required to have substantial knowledge in at least three of the research themes listed above. England, France and Germany were chosen as target markets due to having an advanced HTA system and using PRO data in HTA. While many European countries use PROs in HTA, England, France, and Germany follow distinct and well-established HTA approaches, each with different expectations for PRO evidence. Participant demographics are not presented to preserve anonymity of the KOLs. Recruited participants were not informed of the study sponsor identity until the end of the study to avoid bias. Research responses were anonymized.

### Qualitative interviews and analysis

Semi-structured qualitative interviews of approximately 60-minutes were conducted virtually via Microsoft^®^ Teams to gather feedback on the integration of PRO evidence into HTA submissions. All conversations were audio recorded and transcribed verbatim using the Flatworld Solutions application. Transcripts were checked and amended by Flatworld’s certified proofreaders. One reviewer analyzed the interview transcript, and a second reviewer was responsible for quality control. The transcripts were analyzed using content analysis, key results extracted by a single analyst into Microsoft^®^ Excel for independent review and verification by a data controller, with discrepancies discussed and resolved by consensus. In alignment with General Data Protection Regulation (GDPR), the companies acting as data controllers in this study were Gilead, the study sponsor, and Clarivate, the research agency that designed and conducted the interviews during this research.

## Results

### Use of PRO data in HTA

The availability of guidance on PRO data collection, analysis and reporting in HTA varies across HTA bodies in England, France and Germany, based on the HTA guidance documents identified in the TLR and qualitative interviews (Table 1) [11–28] For example, preferred PRO instruments are specified for HTA submissions in England and France, but not Germany. Most guidance, where available, is country specific, and therefore lacks harmonization across included markets.

**Table 1:** PRO data analytics for HTA in England, Germany and France

		Commonly cited uses	Preferred instruments	Use of PRO data for safety	Missing data threshold	PPD collection	MCID threshold	TTE endpoint definitions	Missing data sensitivity analysis	Multiplicity adjustment
**National Standard** ^**†**^	**England**	• HSU/ economic model • HRQoL, symptoms, function impact • Safety/toxicity/ tolerability • Patient preference/ satisfaction • Natural disease history	Yes (EQ-5D-3 L for HSU elicitation)	Yes (mapped to the EQ-5D-3 L)^‡^	Not defined	No	No	No	No	No
	**Germany**	• HRQoL, symptoms, function impact • Safety/toxicity/ tolerability • Patient preference/ satisfaction	Not defined	Yes (captured by a validated instrument, such as PRO-CTCAE)	Yes (≤ 30%)	Yes (until death or dropout)	Yes (≥ 15% of the scale range)	No	No	No
	**France**	• HRQoL, symptoms, function impact • Safety/toxicity/ tolerability • Natural disease history	Yes (EQ-5D-5 L for HSU elicitation)	No¶	Not defined	No	No	No	No	Yes
**Cross-market**	**Alignment**	• Accepted: disease- and treatment-related HRQoL impact, symptom/function benefits and treatment satisfaction, patient-reported AE/tolerability	• Validated instruments • Direct measurement in trial(s) preferred	• Captured by validated tools in England and Germany	• Missing data negatively impact HTA outcomes	• KOLs suggest increasing use of the rule ‘the more data, the better’ (i.e. minimized data gaps) • Direct measures preferred	-	• No national HTA preference for TTE endpoint definitions; similarities in relevant indications, (oncology, migraine)	• Acceptable methods (e.g. MMRM) are determined on a case-by-case basis (but LOCF is generally not preferred)	-
	**Variation**	• Economic applications of PRO analyses (HSU elicitation) are not within G-BA methods	• Accepted: HRQoL data from trials, literature and KOLs (England). Data from literature rejected in Germany • Increasing importance of patient involvement in France	• Generally not used in France	• Informal threshold (≤ 20% in France, not quantified in England)	• PPD data analysis expected for oncology indications in Germany and England, but not in France	• Non-standardized MCIDs reflecting disease-specific expectations in England and France, but not in Germany	• No defined standard. KOLs note “time-to-deterioration” is favored for progressive diseases	• Variable willingness (but higher in England and France) to use literature-derived data to address gaps	• Generally not considered in England and Germany • In France, multiplicity adjustment is mandatory, requiring HRQoL endpoints to be included in the hierarchical testing and pre-specified statistical analysis plan

Abbreviations: AE, adverse event; CTCAE, Common Terminology Criteria for Adverse Events; EQ-3D, EUROQOL-three dimensions; EQ-5D, EuroQoL-five dimensions; HSU, health state utilities; HRQoL, health-related quality of life; HTA, Health Technology Assessment; ICER, incremental cost-effectiveness ratio; KOL, key opinion leader; LOCF, last observation carried forward; MCID, minimal clinical important difference; MMRM, mixed model for repeated measures; PPD, post-progression data; PRO, patient-reported outcome; TLR, targeted literature review; TTE, time-to-event

† Based on the guidance documents identified in the TLR [11–28] and qualitative interviews

‡ KOLs noted that AEs generally do not substantially influence the ICER and therefore HTA outcomes, unless they significantly impact HRQoL or pose high cost to the healthcare system

¶ Assessment of AEs through clinical trials is the preferred method for subjectively quantifying safety data. PROs can supplement AE data and treatment-related impact on HRQoL

HTA bodies in England, France and Germany require use of validated instruments to collect PRO data [11–14]. Preferred instruments are specified for HTA submissions in England and France, but not Germany (Table 1). In England (NICE), the most frequently reported use of PROs is for elicitation of health state utility values, and NICE specifies a preferred instrument (EQ-5D-3 L) for the utility index. Beyond requirements for economic modeling, NICE does not dictate the PRO instrument but seeks disease-specific measures based on NICE precedent or as advised by clinical experts [29]. An interviewed KOL from England stated that “*PRO data [other than EQ-5D] are looked at as part of the [NICE] clinical effectiveness assessment*,* and indirectly*,* all [PRO-related regulatory guidance] serve as kind of good practice guidelines*,* influencing trial design and ultimately the outcomes and evidence that gets presented to NICE. On the cost-effectiveness assessment*,* none of these have very much impact*,* if any.”*

Generic or disease-specific PRO instruments are accepted in France (HAS), with the latter generally preferred for their specificity. The improvement in medical benefit (Amélioration du Service Médical Rendu [ASMR]) rating scale, a 4-level scale that rates clinical benefit of a product from ‘insufficient’ to ‘important’, is used to compare the technology being evaluated with comparator treatments [30]. HTDs who seek improvement in ASMR ratings in evaluations are advised to use the EQ-5D as this is preferred for cost-effectiveness analyses. Alternative generic measures or disease-specific PROs may be considered if pre-defined in the sponsor’s statistical analysis plan. An interviewed KOL from France noted that HAS applies strict methodological requirements for use of PRO data (for example, double-blinded trials, hierarchical testing), resulting in some PRO data not being considered as it would in other markets “*[Different to the approach of] IQWiG. France has mandatory hierarchical testing…but it is very*,* very difficult to integrate PRO endpoints into hierarchical testing. Several questionnaires are global scores [while manufacturers may seek] to select one or two subdimension scores [for HTA]. I hope that within the JCA [Joint Clinical Assessment in Europe]*,* the French approach will not be adopted because this hierarchical testing is too strict*,* especially for PROs.*”

The EQ-5D visual analog scale (VAS) is accepted by the G-BA in Germany as a measurement of patient-reported morbidity, which focuses on disease burden, but not for HRQoL, which is a broader, multidimensional tool capturing a person’s overall wellbeing across physical, emotional and social domains [31, 32]. Items of the European Organisation for Research and Treatment of Cancer Quality of Life Questionnaire Core 30 (EORTC QLQ-C30) (for use in oncology) may be assessed for morbidity and HRQoL separately. Regarding use of PROs in HTA, a German KOL said “*The general rules [for PRO] are not very different from the EMA level—based on validated instruments*,* a definition for the minimal clinically important difference*,* and so on—so very much like the definitions on the IQWiG and G-BA level. [But IQWiG/G-BA methods documents] are more specific and more relevant to the German assessment process.*”

Cross-market alignment and variation in terms of use of PRO data in HTA is summarized in Table 1.

### Use of PRO data for safety and tolerability assessment

KOLs in each market (England, France and Germany) noted that, although patient-reported adverse event (AE) data could be applied to assess safety and tolerability in HTA, they are not commonly used (Table 1). The KOLs shared that patient-reported AEs could differentiate between similarly efficacious therapies according to their perceived impact on HRQoL, for example due to different tolerability and AE severity (including the relationship to treatment adherence). A KOL from England said “*For the sake of argument*,* if there was a really positive reduction in side effects with a new treatment and that was captured by a HRQoL measure in the pivotal trial*,* then that could offer some positive influence.*” In addition, the KOLs from France noted that PROs are considered suitable for assessing AEs, particularly for subjective AEs (for example, nausea, pain, headache, anxiety), as well as for symptomatic tolerance of drugs, but traditional assessment of safety through direct capture in clinical trials is preferred for subjective AEs with drugs being evaluated by HTA.

### PRO data analytics

#### Missing data threshold

For HTA submissions in Germany, the threshold at which the amount of missing data is acceptable is ≤ 30% (with a per-treatment arm threshold of ≤ 15%), as cited by the KOLs, along with the supporting references [13, 14] (Table 1). Transparency is lacking for the threshold accepted by NICE and HAS; the interviewed KOLs suggested an unquantified and informal preference for a ≤ 20% threshold for missing data may apply. In terms of addressing missing data, in Germany, the mixed model for repeated measures (MMRM) is the preferred method for handling missing data and is accepted provided the missing at random (MAR) assumption holds. The last observation carried forward (LOCF) method may be accepted by IQWiG/G-BA, but it is generally not preferred over other methods (for example, the MMRM) due to doubts around LOCF in scenarios involving fluctuations in HRQoL and symptoms. Use of LOCF and next observation carried backward (NOCB) methods in HAS submissions depends on the context, for example, single-time missing data may be handled with NOCB or LOCF (although LOCF may be inappropriate for situations where patient outcomes change over time). Multiple imputations as primary or sensitivity analyses may be more appropriate for missing data due to loss to follow up. Numerous methods for addressing missing data have been used in NICE HTA submissions, according to interviewed KOLs, including multiple imputations, LOCF, and data imputation by taking averages between available data points. LOCF is not preferred over regression-based methods (as even a minor adjustment in the utility values estimated from LOCF can significantly influence cost-effectiveness outcomes). Scenarios may be examined both with and without imputed data.

#### Post-progression data collection

Interviewed KOLs stated that post-progression data (PPD, PRO data that is collected when a patient’s disease has progressed), should be expected by HTDs to be a requirement for oncology submissions in Germany and England (Table 1). The IQWiG/G-BA specify a preference for data to be collected until patient dropout or death. Interviewed KOLs explained that practical limitations mean that data collection over the full trial period and 30 or 100 days after progression or disease/symptom-relevant timeframes may be accepted on a case-by-case basis. Post-progression analyses using data from external sources (such as published literature) are unlikely to be accepted. For submissions to NICE, a lifetime time horizon is expected, and PPD PRO data collection until death is preferred (and required for economic modeling). Requirements for PPD are not currently transparent but are under discussion in France.

#### Minimal clinically important difference threshold

The threshold for a minimal clinically important difference (MCID) for PROMs is specified for submissions in Germany only (≥ 15% of the PRO scale range) (Table 1). NICE and HAS have non-standardized MCIDs reflecting disease-specific expectations (and therefore no single threshold was mentioned by the interviewed KOLs).

#### Time-to-event endpoints

PROs can be used to define the time from treatment initiation until a predefined decline in a patient’s health status occurs, as measured by a PRO (for example, a specific decrease in a functional score or increase in a symptom score) [33].

There were no identified national HTA preferences for time-to-event (TTE) endpoint definitions or requirements for missing data sensitivity analyses.

#### Multiplicity adjustment

Multiplicity adjustment (a statistical method to control the increased risk of false positive errors when multiple hypotheses or comparisons are tested within a single study [34]) does not play an important role in the HTA processes in Germany and England. In France, although multiplicity adjustment analysis requirements are not PRO-specific, HAS only accepts PRO data that are fully controlled for HTA assessments in line with guidelines.

### Perceived impact of PROs in HTA

Interviewed KOLs from England (*n* = 4) gave variable ratings of low impact, low-to-moderate impact and high impact, based on what they perceived the impact of PRO data to be on HTA decision making (Table 2). The impact of PROs (beyond the use of EQ-5D data in generation of quality-adjusted life years (QALYs]) in HTA decision making by NICE was reported to be variable by disease, and therefore one KOL did not provide a rating. In terms of the impact of PRO data on pricing of evaluated treatments, interviewed KOLs gave ratings of low impact, moderate impact and potential high impact if accompanied by a positive cost-effectiveness analysis. Improvement in HRQoL exerts an indirect influence on NICE decision making, depending on the relative importance of PROs in assessment of specific diseases. Cost-effectiveness requirements must be met to receive a NICE funding recommendation and PRO data do not independently influence reimbursed price negotiation.

**Table 2:**
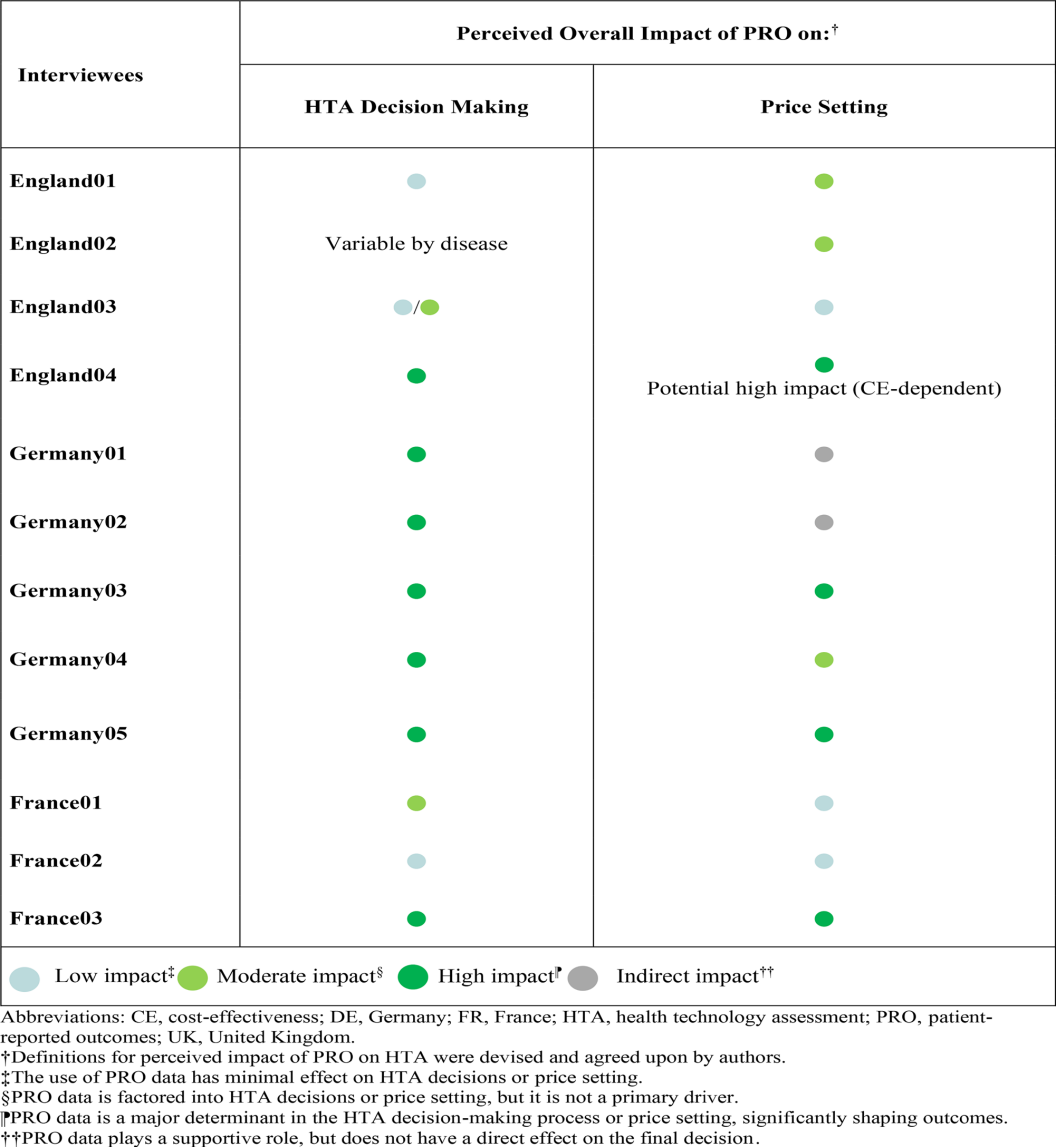
The impact of PROs on HTA decision making and price setting

In Germany, interviewed KOLs (n = 5) gave a rating of ‘high’ based on what they perceived the impact of PRO data to be on HTA decision making (Table 2). In terms of the impact of PRO data on pricing, interviewed KOLs gave ratings of indirect impact, moderate impact and high impact. A meaningful improvement in patient HRQoL can elevate the HTA benefit rating by one step, for example, from ‘non-quantifiable’ to ‘minor’ benefit.’ A ‘considerable’ benefit rating was reported to require clinical efficacy data. The absence of HRQoL data can lower the attainable benefit rating level and therefore negatively impact benefit ratings. Because PROs can have an impact on benefit rating, they can indirectly affect drug pricing. The emphasis in price negotiations is on the overall additional benefit (rather than individual components), alongside prices of comparators, and price in other European markets. However, the KOLs noted that price leverage depends in part on the influence of PROs vs. other endpoints in the indication.

In France, interviewed KOLs (*n* = 3) gave ratings of indirect impact, moderate impact and high impact based on what they perceived the impact of PRO data to be on HTA decision making (Table 2). Ratings of indirect impact and high impact were given in terms of the perceived impact of PRO data on reimbursed price setting in HTA. PRO data can help HTA decision makers to understand the unmet medical needs (for example, burden of rare diseases), potentially elevating the ASMR (a 4-level scale that rates clinical benefit of a product from ‘insufficient’ to ‘important’) [30], and consequently influencing the reimbursement outcome. Interviewed KOLs reported that PRO data demonstrating a significant benefit to patients, beyond primary efficacy measures in the pivotal trial(s), can have moderate-to-high impact on ASMR rating, enhancing price potential.

In addition to the perceived overall impact of PRO data in HTA and/or pricing outcomes, KOLs provided insights into how the impact of PROs in HTA may vary by disease. It was reported that PROs are expected for submissions in highly symptomatic and burdensome conditions, but that the relative importance of PRO to HTA outcomes varies by disease. For example, in oncology, PROs capture aspects of patient experience (for example, relapses, symptoms) to assess disease- and treatment-related impact on HRQoL. For debilitating diseases, PROs were cited as key for symptoms that can be captured through self-reporting, such as pain, functional status and skin itch. KOLs noted the common lack of PRO data in rare diseases and stated that reliance on expert opinion may be necessary (and acceptable) for HTA.

A French KOL noted that PRO data from qualitative research may be accepted by HAS to better understand the disease severity and burden, potentially influencing ASMR rating, but not as evidence of drug efficacy. KOLs in both France and Germany noted that PROs reflecting real-world evidence (RWE) (outside trials) are increasingly important. In addition, German IQWiG/G-BA methodology documentation suggests that for conditional reimbursement/early access, routine data collection for RWE generation is conducted, including data on HRQoL. PROs were perceived by the interviewed KOLs as less relevant for acute diseases, and that there may be a lack of validated PRO instruments in acute diseases.

### Issues with using PRO data in HTA submissions

KOLs cited challenges with using PRO data in HTA that may impact the value perception of PROs for reimbursement. (Table 3). Commonly cited challenges, mentioned by at least one KOL per market, were ‘no/suboptimal PRO data collection/submission (for example, due to value perceptions)’ and ‘selected instruments not fit-for-purpose/PRO benefits not adequately represented in models’.

**Table 3:** Challenges of Using PROs in HTA, According to Interviewed KOLs from England, Germany and France

Most to Least Commonly Cited Challenges in PRO Submissions^†^		
Increasing number of mentions	No/suboptimal PRO data collection/submission (e.g., due to value perceptions)	Selected instruments not fit-for-purpose/PRO benefits not adequately represented in models
	Inappropriate use of PROM or non-validated instrument	Study design (e.g., blinding/endpoint selection) & bias
	Failure to meet statistical/EQ-5D mapping rigor	Missing or insufficient PRO data
	PRO requirement discrepancy among stakeholders (e.g., HTA vs. regulatory agencies)	
	Comparator PRO data not collected/submitted	
	Low awareness of PRO, leading to non-compliance	Real-world applicability of collected data
**KOL quotes**		
England	*“The first issue* [with using PROs in HTA] *is having the right outcome measure in the clinical evidence…NICE has a specific preference for the EQ-5D… I still think there is an issue in some situations where either EQ-5D isn’t being included in the clinical studies or no generic outcome measure that allows you to calculate QALYs is being included. It is rarer*,* but it does still happen. And I think often that is because the trials aren’t just designed for the UK market and you know*,* there are global influences and sometimes the global HQ for the industry doesn’t fully understand how important it is. The other situation is that they look to include some other outcome measures that can be accepted. But I think the difficulties come when companies make that decision without having empirical evidence to justify that choice…The third is where the correct outcome measures have been included in the trials*,* but they are insufficient. So the economic model nearly always has to extrapolate beyond the trial….So often we are have to supplement the evidence that has been collected in the trials with evidence from elsewhere.”*	
Germany	*“For about the last 5–10 years*,* there has been an increase in PRO…We want PRO factoring into assessments*,* but the problem is that PRO studies are not done*,* are not collected*,* or they are collected in an insufficient way. The data quality has not been good. I would say this is improving. On the whole*,* PROs can play a major role in German HTA if collected and analyzed adequately.”*	
France	“*If you have PRO data collected with a poor methodology*,* for example*,* in an open-label trial design or with many missing data*,* it will not be considered. So the impact* [of PRO on HTA decisions] *is dependent on the quality of PRO data.*”	

Interviewed KOLs were from England (*n* = 4), Germany (*n* = 5) and France (*n* = 3). Challenges on the same row were cited equally

Abbreviations: EQ-5D, EuroQoL-five dimensions; HTA, Health Technology Assessment; KOL, key opinion leader; NICE, National Institute for Health and Care Excellence; PRO, patient-reported outcomes; PROM, patient-reported outcome measure; QALY, quality adjusted life year; UK, United Kingdom

Insights on the potential for international HTA harmonization were provided by the interviewed KOLs. Although potential cross-market PRO standards could be supported by Joint EU HTA regulation, interviewed KOLs highlighted that uncertainties and challenges remain. For example, an interviewed KOL from Germany mentioned that the requirements for PRO data in HTA are likely to vary across markets, given that HTA decisions and guidelines are formulated based on the specific needs of individual healthcare systems, making cross-market harmonization challenging.

The intersection of JCA guidance (which aims to harmonize the clinical aspect of HTA for new treatments in EU member states) and PRO data requirements for HTA according to methods guidance and KOL feedback is presented in the Appendix (Supplementary Table 1), and highlights differences between PRO data requirements for HTA in included markets and JCA guidance.

## Discussion

The results of a TLR and semi-structured qualitative interviews with KOLs with HTA- and PRO-related expertise from England, France and Germany indicate that there is insufficient transparency in terms of PRO data requirements by HTA bodies in included markets. For example, preferred PRO instruments are specified for HTA submissions in England and France, but not Germany. Where guidance is available, it is country specific and lacks transparency in terms of how PRO data are weighted in the decision-making process, reflecting a need for greater cross-market harmonization. These findings are consistent with studies reporting the variability of PRO requirements in HTA across countries and the challenges this creates for HTDs when designing trials intended to support HTA decisions [35, 36]. Prior work has underscored the lack of harmonized guidance and the difficulty of aligning with country-specific expectations [35, 36].

Health technology developers and researchers require standardized methodologies for data collection, reporting and analysis of PRO endpoints for HTA. Given the lack of transparency in PRO data requirements by HTA bodies, HTDs face uncertainty around HTA expectations to guide their PRO strategy and analysis methods when planning for HTA submissions. As a result, HTDs may be prevented from coordinating clinical development and HTA strategies, which may negatively impact reimbursement. Recent case studies have demonstrated the importance of including PRO/HRQoL data in HTA, and, in particular, how highly HTA bodies value these data when they are collected according to guidance [5, 37–41].

In this study, interviewed KOLs from Germany perceived the impact of PRO data on HTA decision making to be high. KOL opinion suggests that, in the French setting, PRO data could have an indirect impact on HTA decision making, as PRO data can help HTA decision makers to understand the unmet medical needs (for example, burden of rare diseases). Across countries, KOLs noted that PROs are expected for submissions in highly symptomatic and burdensome conditions, and to a lesser extent for acute diseases, which may not have validated PRO instruments. These insights build on existing interview-based research exploring stakeholder perceptions of PROs in HTA by providing updated, country-specific perspectives [42, 43].

To encourage the collection of PROs in clinical trials, European HTA bodies have an opportunity to issue harmonized requirements for collection, reporting and analysis of PRO data in HTA, given the recent acceptance by the EU council for joint HTA JCA and the ongoing Setting International Standards of Patient-Reported Outcomes and Quality of Life Endpoints in Cancer Clinical Trials – Innovative Medicines Initiative (SISAQOL-IMI) project, which aims to develop standardized methods to analyze and report findings of PRO evidence from oncology trials. However, there are inconsistencies between JCA guidance and national PRO data requirements for HTA in England, France and Germany, further highlighting the challenges faced by HTDs when intending to use PRO data from clinical trials in HTA and the need for harmonization of PRO data requirements across Europe.

The JCA guidance applies only to the clinical, not the economic, assessment of new health technologies, and its relevance and incorporation may vary across the countries considered in this study. In Germany and France, where access and pricing decisions are based on clinical benefit, JCA guidance on PRO data is both applicable and relevant. In England, PRO data are primarily used in economic models to inform cost-effectiveness analyses, which fall outside the scope of the JCA and are conducted independently at the national level. Furthermore, as England is not part of the European Union, JCA guidance does not apply. However, the UK participates in an international HTA collaboration with agencies in Australia, Canada, and New Zealand, aimed at addressing shared methodological challenges including those related to PRO use. This may offer a platform for harmonization of PRO data requirements for HTA. In addition, the Subgroup for the Development of Methodological and Procedural Guidance, a component of EU HTA regulation in the EU, may provide an additional mechanism for harmonizing guidance on the use of PRO data across HTA.

The challenges with using PRO data in HTA identified highlight that there are issues in translating the patient experience into health system decision making. Established conceptual frameworks outline the pathway from patient experiences to measurable outcomes and treatment value [44, 45]. However, the results of this study highlight how gaps in harmonized methodology can disrupt this pathway, limiting the utility of PROs in HTA submissions. Based on challenges associated with PRO submissions cited by the interviewed KOLs, and in alignment with guidance provided in other research studies [46, 47], key considerations for HTDs are summarized in Table 4.

**Table 4:** Challenges Cited by Interviewed KOLs Associated with PRO Submissions and Key Considerations for HTDs

Cited Challenges	Key Considerations for HTDs^†^	
No/suboptimal PRO data collection/submission (e.g., due to value perceptions of PRO data)	Plan submissions using PROs, especially in high-symptom/high-burden conditions	Increase awareness among industry and HTA bodies of the value of PROs in the target indication, where evaluation methods and priorities reflect increased patient engagement, e.g., in PRO measure selection (such as JCA evaluations and national HTA methods), and communicating literature evidence to alleviate concerns around bias of open-label studies
PRO requirement discrepancy among stakeholders (e.g., HTA vs. regulatory agencies)	As regulators emphasize the importance of including patient perceptions of symptomatic AEs in submissions (for reporting safety in interventional trials), MedDRA terminology is preferred. The use of a different terminology standards should be justified by the HTD, for example for the use of oncology-specific questionnaires (e.g. PRO-CTCAE, with a rating for severity/seriousness), per JCA and national HTA guidance (10) To mitigate concerns about use of a subset of selections of the full PRO-CTCAE, individual patient-reported symptomatic AEs should be pre-specified with justification in the study protocol. In addition, using free-text for patient-reported symptomatic AEs would be good practice to capture unexpected or previously unidentified events	
Comparator PRO data not collected/submitted		
Low awareness of PRO, leading to non-compliance		
Selected instruments not fit-for-purpose/PRO benefits not adequately represented in models/inappropriate use of PROM or non-validated instruments	Consider use of both validated generic and disease-specific PRO instruments for submissions As a standard MCID threshold exists only in Germany (and is not planned by JCA (10)), PRO data without context (for example, literature-validated MCID by disease) may be criticized in other markets. Therefore, use of literature-derived data to address gaps, if required, could be considered	
Failure to meet statistical/EQ-5D mapping rigor for using PRO data in HTA		Conduct missing data sensitivity analyses per JCA standards (“as many as appropriate”) (10), and implement rigorous clinical and statistical practices, with clear methods and definitions
Missing or insufficient PRO data	Aim for ≤ 20% missing data to meet both formal (Germany) and informal (England and France) expectations, ideally lower.	
	Plan for PPD analyses as a standard for all oncology submissions	
Real-world applicability of collected data	If using TTE endpoints, the event should be clearly defined, with validated tools for measurement. Baseline values, data collection time points and frequency of assessments should be reported to provide context for change scores	
Study design (e.g., blinding/endpoint selection) and bias	Apply strategies to reduce bias risk in reporting of outcomes (for example, avoid omissions of key measures or timepoints, changing measure scoring, additions of unplanned analyses and discrepancies between the study protocol and the SAP)	

Abbreviations: AE, adverse event; EQ-5D, EuroQoL-five dimensions; HTA, Health Technology Assessment; HTD, health technology developer; JCA, Joint Clinical Assessment; KOL, key opinion leader; PRO, patient-reported outcome; PROM, patient-reported outcome measure; PRO-CTCAE, PRO-Common Terminology Criteria for Adverse Events; MCID, minimally clinical important difference; MedDRA, Medical Dictionary for Regulatory Activities; MMRM, mixed model for repeated measures; PPD, post-progression data; PRO, patient-reported outcome; SAP, statistical analysis plan; TTE, time-to-event

†Considerations according to study authors

There are limitations of this work related to the small sample size of interviewed KOLs. The purpose of conducting qualitative interviews was to gain an in-depth understanding of the experiences, perspectives, and challenges faced by stakeholders regarding PRO use in HTA. The sample was therefore selected for expertise, which resulted in a small sample size. This approach aligns with qualitative methodologies where the goal is to generate insights and deepen understanding rather than generalize findings, and has been used in other studies [48–50]. In addition, a small sample size is often appropriate and sufficient for capturing in depth information and expert point of view [48–50]. However, the authors note that the small purposive sample size, while appropriate for qualitative research, may limit the breadth of perspectives and should be interpreted within its context. In addition, as participants represented England, France and Germany, the results may not apply to PRO data utilization and data analytics of other European markets.

Despite these limitations, this study provides qualitative insights from KOLs with expertise in HTA, presenting up-to-date, country-specific perspectives on the use of PRO data in HTA. It highlights how PRO data may influence decision-making differently across settings, depending on factors such as local unmet needs and disease burden. Further research and discussion in cross-market harmonization is needed, and the authors of this manuscript call for relevant stakeholders to establish channels for this collaborative work.

## Conclusion

There is a lack of transparency and harmonization in terms of the requirements for PRO data collection, analysis and reporting for HTA in England, France and Germany. Enhanced transparency of HTA requirements will help harmonization efforts, and may be supported by the JCA, ultimately facilitating inclusion of the patient voice in a meaningful way for HTA bodies.

## Appendices

### Appendix 1

The participant screener for KOLs with expertise in PRO policy making or assessment of PRO data in HTA submissions in England, France and Germany is presented below:

## Introduction

### General disclosures

Welcome and thank you for your interest in participating in this research. We are conducting market research on behalf of a pharmaceutical company. The purpose of this research is to gain a better understanding of better understanding of the PRO data for HTA analytics/decision-making. The aim of this market research is to gain your views and is not in any way promotional. If you qualify, you will be eligible to participate in a survey and will be compensated for your time and participation. We will ask you a few questions to confirm that you meet the eligibility criteria for participation. This should only take a few minutes of your time.

- The research complies with Insights Association Code of Conduct as well as the BHBIA, ESOMAR, EphMRA’s Legal and Ethical Guidelines.
- Compensation for participation in the study is £210, €250 and €300/hour for participants from the UK, France and Germany, respectively. This compensation is not intended to reward you for use of any products which may be mentioned in the study or induce you to use any such products but, rather, is in exchange for your time and participation in this market research and to understand your views.
- During this study and in this screener, you may be asked questions which, if answered, may require you to disclose information that you or your organization considers to be confidential. Please alert us if this happens or do not provide this confidential information. It is not our intent to ask you to disclose such information.
- By participating in this study, you acknowledge that any proprietary information regarding products and product development and other trade secrets and know-how may not be disclosed, and you agree to hold all such information confidential and not disclose it to any third party or use it for any other purpose whatsoever.
- Any information you provide us with during the course of this market research will be treated as confidential and not attributed to you as an individual. Rather, the information you provide will be combined with feedback from others like yourself and reported in the aggregate to the sponsor of this market research.
- You have the right to refuse to answer questions and may withdraw from the market research at any time, and you will have the right to withhold information, i.e., not answer a question, should you wish to.
- The WCG Institutional Review Board (IRB) approved a request that this research is exempt from an IRB review.

### Adverse events or product complaints

We are now being asked to pass on to our client details of adverse events that are mentioned during the course of market research interviews. Although this is a market research interview and what you say will, of course, be treated in confidence, should you raise during the discussion an adverse event in a specific patient, we will need to report this to our client, even if it has already been reported by you. Except as otherwise required by law or applicable regulation, everything else you say during the course of the interview will continue to remain confidential.

Do you provide consent to this?

**Table:** 

Yes	□	Continue
No	□	**Terminate**

### Data privacy and protection disclosure including cross border data transfer

Personal data about you will be collected and used for the purposes of the market research study. Such personal data may include your name, your contact information, your professional background and qualifications, and your opinions. We will only collect or process data that is absolutely necessary to conduct the study, and not all aforementioned categories will be applicable to all studies. To the extent your personal data is collected or processed in conjunction with the market research study, it may be transferred to third parties assisting with the study, such as a survey programmer, and will also be transferred to the United States. In such cases, the necessary measures will be taken to ensure the safety of your personal data in accordance with applicable data protection laws. You can find out more about our privacy practices around processing and transferring data by reading the Clarivate’s Privacy Policy, which is available at https://clarivate.com/privacy-center/notices-policies/privacy-policy/.

Do you agree to the collection, processing, and transfer of the personal data necessary to participate in this study?

**Table:** 

Yes	□	Continue
No	□	**Terminate**

### Data controller

In accord with the EU General Data Protection Regulation], we are required to reveal the names of all entities defined as “Data Controllers”. For this study, the pharmaceutical company that commissioned the research, the marketing research agency and the recruiters are data controllers. We would prefer to reveal the names of the data controllers at the end of the survey or interview to prevent bias in the responses provided. Is it acceptable to you to delay knowing the data controllers until the end of the interview / survey?

**Table:** 

Yes	□	Continue
No	□	**Terminate**

Please note you can withdraw your consent to participate at any time.

## Recording

For this research we would like to audio record your interview. The interview recordings and any other market research content you provide may be shared with Clarivate‘s subcontractors for purposes of the market research study, who will respect the confidentiality of all information exchanged. Clarivate will not reveal your name and will ensure that you are not personally identifiable to anyone who receives the recordings.

You may withdraw this consent at any time.

Do you agree to recording of the interview for note taking purposes?

**Table:** 

Yes	□	Continue
No	□	**Terminate**

### Permission to be re-contacted

For this research we may need to re-contact you if, following the study, we need clarification on any responses. Do we have your permission for Clarivate to re-contact you if necessary?

**Table:** 

Yes	□	Continue
No	□	**Continue**

### Screener questions

Your answers to the following questions will help us ensure your expertise aligns with our needs for this study:

1. Are you an (ex-)payer/payer-advising key opinion leader (KOL) who has in-depth knowledge of patient-reported outcome (PRO) data and analytics utilized in national health technology assessment (HTA) and/or pricing and reimbursement (P&R) evaluations (e.g., a former member and/or current advisor to the national HTA body, or relevant subcommittees, that inform drug funding decision-making)?

**Table:** 

Yes	□	Continue
No	□	**Terminate**

2. Is your expertise in cross-market PRO data and analytics in funding decisions, or more from a country-specific perspective?

**Table:** 

Cross-market (please specify)	□	Continue
Country-specific	□	**Continue**

3. Have you advised on/participated in drug evaluations, in particular as they relate to the PRO data analytics for reimbursement submissions, in the past 5 years?

**Table:** 

Yes	□	Continue
No	□	**Terminate**

4. Please rate your level of understanding for each of the following guidance and resources for PRO data analytics listed below.

**Table:** 

Existing guidance and resources for PRO	Ratings: - Significant understanding - Some understanding - Minimal understanding - No understanding
FDA’s Patient-Focused Drug Development (PFDD)	**Continue**
Standard Protocol Items: Recommendations for Interventional Trials (SPIRIT)-PRO	**Continue**
EMA Appendix 2 on the use of PRO measures in oncology studies	**Continue**
PROTEUS Consortia resources (guidelines on rigor and utility of PRO endpoints in clinical trials)	**Continue**
Other (please specify)

5. Are you comfortable in discussing the following topics?

**Table:** 

Themes	Record yes/no	
1. **General integration of PROs in HTA** (e.g. major hurdles incorporating PRO in HTA, impact of PRO on final HTA decision/recommendations)		**Continue if respondent says “Yes” for Theme 1 and says “Yes” for at least 2 sub-themes from Themes 2/3**
2. **PRO collection and report**		
• Preferred instruments		
• Missing data threshold		
• Post-progression data collection		
3. **PRO data analysis**		
• Clinically meaningful change threshold		
• Preference for time-to-event endpoint definitions		
• Missing data sensitivity analysis		
• Multiplicity adjustment

6. Are you, or is any member of your household/family affiliated pharmaceutical company or healthcare manufacturer, as a clinical investigator, consultant, researcher, or in any other capacity?

**Table:** 

Yes	□	Continue
No	□	Continue

Participant background is presented below:

**Table:** 

Market	Expert profiles	Type	PRO-expertise	Advised on or participated in HTA evaluations	Understanding of existing PRO guidance (e.g. FDA PFDD, SPIRIT-PRO, EMA Appendix 2, PROTEUS)	Self-reported understanding of PRO concepts in the HTA process		
						General integration of PROs in HTA	PRO collection and report	PRO data analysis
**UK**	UK01	Academia	Country-specific	✓	Minimal	✓	3/3	4/4
	UK02	Ex-payer advisor	Country-specific	✓	No	✓	3/3	4/4
	UK03	Ex-payer advisor	Cross-market	✓	Some	✓	2/3	2/4
	UK04	Academia	Country-specific	✓	Some	✓	3/3	4/4
**Germany**	DE01	Ex-payer	Country-specific	✓	No	✓	3/3	4/4
	DE02	Academia	Country-specific	✓	Some	✓	3/3	4/4
	DE03	Ex-payer	Country-specific	✓	Some	✓	3/3	4/4
	DE04	Ex-payer	Country-specific	✓	Some	✓	3/3	4/4
	DE05	Academia	Country-specific	✓	Some	✓	3/3	4/4
**France**	FR01	Ex-payer/ academia	Cross-market	✓	Some	✓	3/3	4/4
	FR02	Ex-payer/ academia	Country-specific	✓	Some	✓	3/3	4/4
	FR03	Ex-payer/ academia	Country-specific	✓	Some	✓	3/3	4/4

### Appendix 2

The intersection of JCA guidance and PRO data requirements for HTA according to methods guidance and KOL feedback is presented in Supplementary Table 1.

## Supplementary Information

Below is the link to the electronic supplementary material.

Supplementary Material 1

Supplementary Material 2

## Data Availability

The datasets used and/or analysed during the current study are available from the corresponding author on reasonable request.

## Abbreviations

AE: Adverse event
ASMR: Amélioration du Service Médical Rendu
BHBIA: British Healthcare Business Intelligence Association
COREQ: Consolidated criteria for reporting qualitative research
EORTC QLQ-C30: European Organisation for Research and Treatment of Cancer Quality of Life Questionnaire Core 30
EQ-5D: EuroQoL-five dimensions
EPHMRA: European Pharmaceutical Market Research Association
EUnetHTA: European Network for Health Technology Assessment
G-BA: Gemeinsamer Bundesausschuss
GDPR: General Data Protection Regulation
HAS: Haute Autorité de Santé
HRQoL: Health-related quality of life
HTA: Health technology assessment
HTDs: Health technology developers
IGWiG: Institut für Qualität und Wirtschaftlichkeit im Gesundheitswesen
JCA: Joint Clinical Assessment
KOLs: Key opinion leaders
LOCF: Last observation carried forward
MAR: Missing at random
MCID: Minimal clinically important difference
MMRM: Mixed model for repeated measures
NICE: National Institute for Health and Care Excellence
NOCB: Next observation carried backward
PICO: Population, intervention, comparison and outcome
PHI: Protected health information
PPD: Post-progression data
PRO: Patient-reported outcome
QALYs: Quality-adjusted life years
QoL: Quality of life
RWE: Real-world evidence
SISAQOL-IMI: Setting International Standards of Patient-Reported Outcomes and Quality of Life Endpoints in Cancer Clinical Trials – Innovative Medicines Initiative
TLR: Targeted literature review
TTE: Time-to-event
VAS: Visual analog scale
WCG IRB: Western Institutional Review Board-Copernicus Group Institutional Review Board
