# Supplementary material for: Requirements for patient-reported outcomes and data analytics in health technology assessment in England, France and Germany, and the need for methods harmonization across European markets: a qualitative interview study
Source: J Patient Rep Outcomes. 2026 Apr 3;10:80. doi: 10.1186/s41687-026-01059-4 (PMC13172063; doi:10.1186/s41687-026-01059-4)
Supplement: Supplementary file 1 — Supplementary Material 1 [file 41687_2026_1059_MOESM1_ESM.docx]

**Table 1. Completed COREQ checklist**

| **Section/Topic** | **Item No** | **Checklist item** | **Response** |
| --- | --- | --- | --- |
| **Domain 1: Research team and reﬂexivity** | | | |
| Personal Characteristics | | | |
| Interviewer/facilitator | 1 | Which author/s conducted the interview or focus group? Interviewer/facilitator | Clarivate team. OYW, LD and SK. |
| Credentials | 2 | What were the researcher’s credentials? E.g. PhD, MD | OYW: MPH |
| Occupation | 3 | What was their occupation at the time of the study? | Consultants at Clarivate |
| Gender | 4 | Was the researcher male or female? | Researchers are all female |
| Experience and training | 5 | What experience or training did the researcher have? | OYW, LD and SK have undertaken training in qualitative research methodologies and have previous experience of this methodology |
| Relationship with participants | | | |
| Relationship established | 6 | Was a relationship established prior to study commencement? | No prior relationship was established between the researchers and participants |
| Participant knowledge of the interviewer | 7 | What did the participants know about the researcher? e.g. personal goals, reasons for doing the research | Participants knew where the researchers worked and the purpose of the research |
| Interviewer characteristics | 8 | What characteristics were reported about the interviewer/facilitator? e.g. Bias, assumptions, reasons and interests in the research topic |  |
| **Domain 2: study design** | | | |
| Theoretical framework | | | |
| Methodological orientation and Theory | 9 | What methodological orientation was stated to underpin the study? e.g. grounded theory, discourse analysis, ethnography, phenomenology, content analysis | The study is a qualitive interview study |
| Participant selection | | | |
| Sampling | 10 | How were participants selected? e.g. purposive, convenience, consecutive, snowball | Potential interviewees were screened and recruited with purposive sampling, based on their knowledge of and experience in Health Technology Assessment |
| Method of approach | 11 | How were participants approached? e.g. face-to-face, telephone, mail, email | By email |
| Sample size | 12 | How many participants were in the study? | 12 participants (from England, n=4; France, n=3; and Germany n=5) |
| Non-participation | 13 | How many people refused to participate or dropped out? Reasons? | Number of refusals were not recorded |
| Setting of data collection | 14 | Where was the data collected? e.g. home, clinic, workplace | Interviews were conducted virtually via Microsoft^®^ Teams |
| Presence of non-participants | 15 | Was anyone else present besides the participants and researchers? | No |
| Description of sample | 16 | What are the important characteristics of the sample? e.g. demographic data, date | Participant demographics are not presented to preserve anonymity of recruited interviewees |
| Data collection | | | |
| Interview guide | 17 | Were questions, prompts, guides provided by the authors? Was it pilot tested? | Interview questions were provided by the authors. It was not pilot tested |
| Repeat interviews | 18 | Were repeat interviews carried out? If yes, how many? | No |
| Audio/visual recording | 19 | Did the research use audio or visual recording to collect the data? | Interviews were audio recorded |
| Field notes | 20 | Were ﬁeld notes made during and/or after the interview or focus group? | N/A |
| Duration | 21 | What was the duration of the interviews or focus group? | 60 minutes |
| Data saturation | 22 | Was data saturation discussed? | N/A |
| Transcripts returned | 23 | Were transcripts returned to participants for comment and/or correction? | N/A |
| **Domain 3: analysis and ﬁndings** | | | |
| Data analysis | | | |
| Number of data coders | 24 | How many data coders coded the data? | 1 |
| Description of the coding tree | 25 | Did authors provide a description of the coding tree? | The coding framework was based on discussion guide themes |
| Derivation of themes | 26 | Were themes identiﬁed in advance or derived from the data? | Themes were derived from results of a TLR in advance then used to inform the interview discussion guide |
| Software | 27 | What software, if applicable, was used to manage the data? | Microsoft^®^ Excel |
| Participant checking | 28 | Did participants provide feedback on the ﬁndings? | No |
| Reporting | | | |
| Quotations presented | 29 | Were participant quotations presented to illustrate the themes / ﬁndings? Was each quotation identiﬁed? e.g. participant number | Yes. Quotations were not linked to interviewees |
| Data and ﬁndings consistent | 30 | Was there consistency between the data presented and the ﬁndings? | Study authors aimed to report study findings in a clear, informative manner in order to accurately reflect the data that have been collected |
| Clarity of major themes | 31 | Were major themes clearly presented in the ﬁndings? | Yes, using subsections in the results section |
| Clarity of minor themes | 32 | Is there a description of diverse cases or discussion of minor themes? | All findings are discussed in the results sections |
