# Supplementary material for: Requirements for patient-reported outcomes and data analytics in health technology assessment in England, France and Germany, and the need for methods harmonization across European markets: a qualitative interview study
Source: J Patient Rep Outcomes. 2026 Apr 3;10:80. doi: 10.1186/s41687-026-01059-4 (PMC13172063; doi:10.1186/s41687-026-01059-4)
Supplement: Supplementary file 2 — Supplementary Material 2 [file 41687_2026_1059_MOESM2_ESM.docx]

**Supplementary Table 1: Intersection of requirements for PRO data analytics in HTA and JCA guidance**

|  | | **Preferred instruments** | **Missing data threshold** | **Use of PRO data for safety and tolerability assessments** | **Post-progression data collection** | **Clinically meaningful change threshold** | **Preference for TTE endpoint definitions** | **Missing data sensitivity analysis** | **Multiplicity adjustment** |
| --- | --- | --- | --- | --- | --- | --- | --- | --- | --- |
| **National standard** | **England^†^** | Yes (EQ-5D-3L for HSU elicitation) | Not defined | Yes (mapped to the EQ-5D-3L)^‡^ | No | No | No | No | No |
|  | **Germany** | Not defined | Yes (<30%) | Yes (captured by a validated instrument, such as PRO-CTCAE) | Yes (until death or dropout) | Yes (≥15% of the scale range) | No | No | No |
|  | **France** | Yes (EQ-5D-5L for HSU elicitation) | Not defined | No^⁋^ | No | No | No | No | No |
| **JCA guidance, EU** | **National standard** | Yes (safety reporting) | Not defined | Yes (MedDRA terminology preferred) | Yes (until death) | No | Yes (TTEs are accepted but not specified) | Yes (HTD to provide SA) | No |
|  | **Relevant recommendations** | **General**   - MSs to choose outcomes based on their own needs for decision making. - Recommendations from well-established COS to be considered for select outcomes   **Instruments:**   - Validated generic or disease-specific instruments. | - The acceptability of missing data is subject the interpretation of the impact of the amount of missing data by MSs. Therefore, reports should highlight the uncertainty with respect to the amount, as well as handling of, missing data | - For reporting safety in clinical trials, MedDRA terminology is preferred. PRO-CTCAE is accepted (with rating for severity/seriousness) | - Requirements for PPD collection are not defined in current guidance. - The MSCG notes: Timing of assessment depends on the intervention and the research question. Outcomes should be long-term or final where possible. - Depending on the research question, short-term outcomes may also be relevant, e.g. symptoms, HRQoL and AEs | - Focus is on responder analysis (pre-specified as part of the primary analysis for each outcome measure) - HTD must describe the measurement scale - Along with results expressed according to a responder definition (summary measure, effect measure), results should be expressed using the original qualitive scale | - Treatment response can be reported as morbidity events or in terms of “TTE” (for occurrence of irreversible binary events) or as the change in clinical status or symptoms. The “event” must be defined, and validated tools should be used. Time points and frequency of assessments should be reported along with baseline values | - SAs are not required for every PICO question and outcome. The HTD should provide as many SAs as appropriate, according to good clinical and statistical practices, along with a clear definition of their purpose and underlying assumption(s) - Focus should also be given to ICEs (to precisely define the treatment effect that is to be estimated). | - MSs may assess evidence from individual studies within the framework of the original study SAP and/or of assessing a statistical summary (or evidence synthesis) of one or several studies within the framework of a SR. The approaches impact the way in which MSs consider methodological issues, e.g. multiplicity and subgroup, sensitivity and post hoc analyses. It is not the intent of JCA guidance to endorse a particular approach but enable MSs to draw conclusions |

Abbreviations: AE, adverse event; COS, core outcome set; CTCAE, Common Terminology Criteria for Adverse Events; EU, European Union; EQ-5D, EuroQoL-five dimensions; HTA, health technology assessment; HTD, health technology developer; HSU, health state utilities; HRQoL, health-related quality of life; HTA, Health Technology Assessment; HTD health technology developer; ICE, intercurrent events; JCA, Joint Clinical Assessment; KOL, key opinion leader; MedDRA, Medical Dictionary for Regulatory Activities; MS, member state; MSCG, member state coordination group; PICO, patient/population, intervention, comparison and outcomes PPD, post-progression data; PRO, patient-reported outcome; SA, sensitivity analysis; SAP, statistical analysis plan; SR, systematic review; TTE, time-to-event.
†JCA guidance does not apply to England as it is not a member state of the EU.
‡KOLs noted that AEs generally do not substantially influence the ICER and therefore HTA outcomes, unless they significantly impact HRQoL or pose high cost to the healthcare system.
⁋Assessment of AEs through clinical trials is the preferred method for subjectively quantifying safety data. PROs can supplement AE data and treatment-related impact on HRQoL.
